# Exploring Astrocyte-Mediated Mechanisms in Sleep Disorders and Comorbidity

**DOI:** 10.3390/biomedicines11092476

**Published:** 2023-09-07

**Authors:** Yujuan Li, Mengxin Que, Xuan Wang, Gaofeng Zhan, Zhiqiang Zhou, Xiaoxiao Luo, Shiyong Li

**Affiliations:** 1 Department of Anesthesiology, Hubei Key Laboratory of Geriatric Anesthesia and Perioperative Brain Health, Wuhan Clinical Research Center for Geriatric Anesthesia, Tongji Hospital, Tongji Medical College, Huazhong University of Science and Technology, Wuhan 430074, China; yujuanli@hust.edu.cn (Y.L.); m202276309@hust.edu.cn (M.Q.); d202182020@hust.edu.cn (X.W.); gfzhan@tjh.tjmu.edu.cn (G.Z.); zqzhouhustjmz@hust.edu.cn (Z.Z.); 2 Department of Oncology, Tongji Hospital, Tongji Medical College, Huazhong University of Science and Technology, Wuhan 430074, China

**Keywords:** astrocyte, sleep disorders, comorbidity, neurodegenerative diseases

## Abstract

Astrocytes, the most abundant cells in the brain, are integral to sleep regulation. In the context of a healthy neural environment, these glial cells exert a profound influence on the sleep-wake cycle, modulating both rapid eye movement (REM) and non-REM sleep phases. However, emerging literature underscores perturbations in astrocytic function as potential etiological factors in sleep disorders, either as protopathy or comorbidity. As known, sleep disorders significantly increase the risk of neurodegenerative, cardiovascular, metabolic, or psychiatric diseases. Meanwhile, sleep disorders are commonly screened as comorbidities in various neurodegenerative diseases, epilepsy, and others. Building on existing research that examines the role of astrocytes in sleep disorders, this review aims to elucidate the potential mechanisms by which astrocytes influence sleep regulation and contribute to sleep disorders in the varied settings of brain diseases. The review emphasizes the significance of astrocyte-mediated mechanisms in sleep disorders and their associated comorbidities, highlighting the need for further research.

## 1. Introduction

Sleep plays a vital role in all animal species possessing a nervous system that has been extensively researched [1]. Nonrapid eye movement (NREM) sleep, comprising three stages (N1, N2, and N3), is characterized by a progressive deepening of sleep and is essential for physical restoration, including tissue repair and immune system strengthening. Rapid eye movement (REM) sleep, distinguished by rapid eye movements and increased brain activity, is the phase where vivid dreaming occurs and is vital for emotional regulation and cognitive processing. The cyclical alternation between these two distinct sleep stages forms the architecture of a healthy sleep pattern, with each stage fulfilling unique physiological and psychological functions. Inadequate or disrupted sleep significantly impacts well-being, hindering cognitive acuity and increasing susceptibility to a wide range of physical and mental disorders [2]. The prevalence of sleep-related disorders, such as insomnia, narcolepsy, and obstructive sleep apnea syndrome (OSAS), varies, yet all of them impose a significant burden on global health [2]. Insomnia, the most common sleep disorder, is estimated to affect up to 30% of adults at some point in their lives, and chronic insomnia affects approximately 10% of adults [3]. OSAS, a prevalent sleep disorder, affects approximately 14% of men and 5% of women in mild cases, and 13% of men and 6% of women in moderate-to-severe cases [4]. Restless legs syndrome (RLS) is a common sleep disorder that is estimated to affect approximately 5–15% of the population, with the incidence increasing with age [5]. Furthermore, sleep deprivation or restriction triggers physiological and neurobehavioral issues [6]. Epidemiological investigations consistently indicate a correlation between inadequate sleep and metabolic dysfunction [7], cardiovascular diseases [8], neurodegenerative diseases [9], psychiatric symptoms [10], and mortality.

Astrocytes, the most prevalent cells in the brain, exhibit a higher astrocyte-to-neuron ratio in human brains (~1–1.4:1) compared to rodents (~0.4:1) [11]. Astrocytes are widely recognized for their multiple functions in maintaining neurotransmitter and ionic homeostasis, modulating synaptic and neuronal activity, and upholding the integrity of the blood-brain barrier (BBB). Moreover, astrocytes are emerging as crucial contributors to the regulation of sleep-wake cycles, both locally and within dedicated neural circuits. These cells dynamically modulate their activity across the sleep-wake continuum, engaging in intricate intracellular signaling pathways that may encode sleep need and exocytose sleep-inducing molecules that influence brain activity and sleep architecture [12]. Studies employing transgenic animals, optogenetics, chemogenetics, and advanced molecular assays have revealed that astrocytes interact with neurons and other glial cells to mediate circadian control [13], modulate sleep-wake behavior through Ca^2+^ activity [14], and even influence sleep patterns through genes known to regulate innate immune responses [15]. The evidence that astroglial sleep mechanisms are preserved across both mammalian and invertebrate species emphasizes the fundamental significance of astrocytes in sleep regulation. Beyond sleep, astrocytes may also have critical roles in the pathogenesis and regulation of neurodegenerative diseases and neurodevelopmental disorders [16]. Although there are a few pioneering studies revealing the essential role of astrocytes in sleep regulation, their involvement in sleep is yet to be fully elucidated, particularly in the context of normal sleep and comorbid sleep disorders.

This review will first provide a summary of the contribution of astrocytes to the regulation of sleep homeostasis. Subsequently, we will discuss neurodegenerative diseases and other pathological conditions, which are often complicated by comorbid sleep disturbances. Subsequently, we will explore the underlying mechanisms through which astrocytes might influence sleep-related comorbidities. The insights gained from this discussion may pave the way for the development of potential treatment strategies for sleep comorbidities.

## 2. The Role of Astrocyte in Regulating Sleep

**Figure 1:**
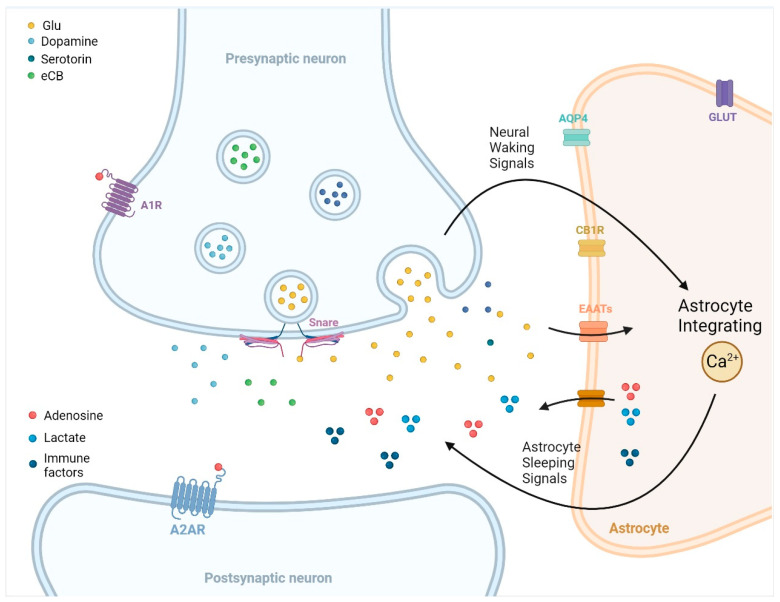
Possible neuronal-astrocyte feedback model for sleep homeostasis. The proposed model consists of three key components: neuronal waking signals that reflect the need for sleep, an astrocyte integrator of the neuronal waking signals, and feedback molecules derived from astrocytes. During periods of wakefulness, certain neurotransmitters and neuromodulators, such as glutamate, dopamine, serotonin, and endocannabinoids, build up. These compounds can influence astroglial ion channels or engage with G-protein-coupled receptors or membrane transporters, leading to alterations in astroglial Ca^2+^ activity. It is theorized that Ca^2+^ is a central component in how astroglia interprets these signals from neurons. As sleep deprivation increases, oscillations in astroglial Ca^2+^ levels are observed, which are believed to be part of the body’s way of counteracting sleep deficit [26]. Furthermore, Ca^2+^ is instrumental in the astroglial secretion of substances that induce sleep. Astroglia releases sleep-inducing signals such as adenosine, lactate, and immune factors. This feedback loop between neurons and astrocytes is thought to be widespread in the brain, influencing both localized sleep and more generalized sleep patterns [27]. AQP4, Aquaporin 4; A1R, adenosine receptors 1; A2AR, adenosine A2A receptor; CB1R, cannabinoid receptor type 1; eCB, endocannabinoids; EAATs, excitatory amino acid transporters; GLUT, glucose transporters, Snare, soluble NSF attachment protein receptors.

The dichotomy of non-REM and REM sleep, each with distinct characteristics, forms the rhythm of our nightly rest. Non-REM sleep serves as a sanctuary for physical restoration, while REM sleep, known for dreams, bursts forth with mental arousal. Although the mechanisms orchestrating this dance between sleep stages have remained elusive, emerging from this complexity is the role of astrocytes, star-shaped glial cells now recognized as pivotal in the sleep-wake cycle. Astrocytes emerged as early as 1895, when Cajal proposed that astrocytes acted as physical barriers that interrupted synaptic transmission during sleep [17]. Since then, a growing body of evidence has suggested that astrocytes are involved in regulating sleep-wake cycles and maintaining homeostasis [18,19,20,21]. Studies have demonstrated that the astrocytic transport or gliotransmission of sleep-inducing molecules modulates sleep homeostasis [22,23,24]. Astrocytic intracellular signaling pathways such as calcium (Ca^2+^) and cyclic adenosine monophosphate (cAMP) have also been discovered to influence sleep [18,25]. A particular model (Figure 1) suggests that astrocytes sense neuronal waking cues emitted during alertness, process these cues through shifts in internal Ca^2+^ levels, and subsequently suppress these wakefulness signals, leading to prolonged sleep durations. Here, neuronal waking signals refer to neurotransmitters and neuromodulators emitted by wake-promoting neurons, while astrocytic sleeping signals encompass molecules such as adenosine, lactate, and certain cytokines, while astrocyte integrating describes the capability of astrocytes to process these cues through internal Ca^2+^ level shifts. The exploration of astrocytes invites us into a world where intricate interplay unfolds, guided by these unsung heroes of the neural orchestra. Further details on these findings will be discussed in the following sections.

### 2.1. Somnogenic Molecules from Astrocyte

#### 2.1.1. Adenosine

More than half of the brain’s energy expenditure is devoted to cognitive functions associated with awareness [28]. At the cellular level, neuronal activity accounts for the majority of adenosine triphosphate (ATP) consumption in the brain. Adenosine is, in fact, a byproduct of ATP metabolism that modulates neuronal excitability. Research by Olivier Pascual and colleagues demonstrated that in a mouse model of selective expression of a dominant-negative SNARE (dnSNARE), employed to impair exocytosis in astrocytes, there was decreased extracellular accumulation of adenosine both in situ and in vivo [29]. This selective expression of astrocytes led to the mitigation of tonic adenosine-mediated presynaptic inhibition of excitatory synaptic transmission. Hypothetically, when paired with an astrocyte-specific inducible system, it could be possible to switch gliotransmission on or off in vivo [29,30]. While research utilizing optogenetic stimulation and genetic manipulation has reported increases in slow wave activity (SWA) and REM sleep during the stimulation of astrocytes in the posterior hypothalamus of mice [31], some researchers have expressed reservations, suggesting that optogenetic stimulation may not be the most optimal method to activate astrocytes [32]. Findings from Halassa and colleagues, who measured SWA in the electroencephalogram (EEG) during nonrapid eye movement sleep (NREMS), showed that the normal accumulation of NREM SWA was reduced in dnSNARE mutant mice. This study also found that the typical compensatory increases in NREMS time post sleep deprivation were notably attenuated [23]. Subsequent studies on the dnSNARE mutant mice revealed that sleep deprivation disrupted the usual changes in brain adenosine concentrations [33]. It is worth noting that, while adenosine usually accumulates during wakefulness, research indicates that extracellular adenosine concentration does not increase during sleep deprivation. These results highlight the complexity of adenosine regulation during sleep and wake cycles, suggesting that sleep deprivation may directly affect adenosine tone [34,35].

#### 2.1.2. Lactate

The astrocyte-neuron lactate shuttle (ANLS), a process whereby the transportation of lactate from astrocytes to neurons is facilitated during periods of elevated synaptic activity, has been established [36]. This concept posits that neurons depend on lactate as their preferred metabolite, which casts astrocytes as crucial suppliers of energy demand [36]. Changes in extracellular lactate concentration are dictated by the sleep-wake cycle; they accumulate during wakefulness and decrease during sleep [37]. Following the hypothesis that neuron-released glutamate is taken up by astrocytes, several studies have investigated this theory by disrupting the glutamate metabolism of astrocytes. These investigations have demonstrated that the inhibition of the astrocyte glutamate transporter in mice can cause sleep disturbances [38], and interrupting the conversion of astrocyte glutamate to glutamine affects neuronal activity and sleep homeostasis in mice [39]. Furthermore, gap junctions such as connexin 43, which form part of the astrocytic syncytium, also play a vital role in supplying lactate to neurons in the brain of mice [40]. In this context, a clinical trial found that THN102, a drug that targets glial connexin activity to modulate the effects of modafinil, could positively affect vigilance and working memory in healthy subjects, providing promising insights for the management of hypersomnolence disorders [41].

#### 2.1.3. Dopamine and Serotonin

Astrocytes have a significant role in promoting sleep by modulating neurotransmitters such as dopamine and serotonin. Norepinephrine, by orchestrating network formation between neurons and astrocytes, plays a key role in neural development [42]. It is established that glial cells metabolize monoamines to maintain sleep homeostasis [43]. A novel study highlighted that astrocytes in the ventral periaqueductal gray could modulate arousal in mice via noradrenergic transmission at α1-adrenergic receptors [44]. In *Drosophila*, the gene *AANAT1*, expressed by astrocytes and specific neurons in the brain, acetylates and inactivates monoamine. When *AANAT1* expression in astrocytes (but not neurons) was silenced, the flies increased their diurnal recovery sleep following overnight sleep deprivation [45]. This result underscores the pivotal role of astrocytes in modulating monoamine and maintaining homeostatic sleep.

#### 2.1.4. Immune Factors and Cytokines

In the realm of immune factors and cytokines, the astrocyte-mediated inflammatory pathway also significantly contributes to sleep regulation. Specifically, astrocytic tumor necrosis factor (TNFα) is an essential regulator of sleep. Following sleep deprivation, TNFα stimulates NREMS and facilitates sleep homeostasis in mice [46], primarily achieved through the regulation of neural activity and synaptic plasticity within sleep circuits [47,48]. A recent study demonstrated that knocking down *Eiger*, a *Drosophila* TNFα homolog, could substantially decrease sleep duration, implying a role for TNFα in sleep homeostasis [49]. Further research confirmed that a TNFα polymorphism, which reduces TNFα function, can predict resilience to sleep deprivation in healthy adults [50]. Astrocyte-produced Interleukin-1 (IL-1) can also enhance SWA in the rat’s sleep EEG [51], and it has been suggested that astrocytes, by secreting molecules such as IL-33, may regulate microglial phagocytosis during sleep in an IL-33 knockout mouse model [52]. Further investigations have revealed intriguing connections between astrocytes and REM sleep. Specifically, transgenic mouse lines expressing interleukin-1 receptor 1 (IL1R1) solely in CNS astrocytes exhibited increased REM sleep [53]. Additionally, astrocyte-derived nitric oxide synthase (NO/iNOS) production has been found to ensure the adequate maintenance of REM sleep in adult rats [54]. Collectively, these findings substantiate the notion that astrocytes have a crucial role in the regulation of sleep and sleep homeostasis.

### 2.2. Astrocyte-Associated Receptors

Studies employing inducible manipulations of different G-protein-coupled receptors (GPCRs) with DREADDs (designer receptors exclusively activated by designer drugs) in mice have highlighted the integral role astrocytes play in sleep. Specifically, noradrenergic α1 receptors and muscarinic acetylcholine receptors can facilitate the Gq/phospholipase C cascade in mice [55,56,57], which function through the activation of IP_3_-activated type 2 receptors (IP_3_R2) on the endoplasmic reticulum (ER). This activation triggers a release of Ca^2+^ from the ER, subsequently raising astrocyte intracellular Ca^2+^ levels in mice [58,59,60]. Activation of Gi-DREADDs in the cortex increased NREM SWA without influencing NREMS duration, while activating cortical astrocytic Gq-DREADDs had the converse effect. This shows that an increase in calcium can be mediated through two distinct GPCR signaling pathways [25], suggesting that astrocytes modulate varied facets of natural sleep via separate signaling mechanisms. Of note, both Gi and Gq signaling pathways can augment intracellular calcium levels in astrocytes in mice [56,61].

As alluded to earlier, astrocytes primarily exert their effects by releasing adenosine, a metabolite of ATP. Once released, it binds G-protein-coupled receptors A1 and A2 (mostly A2A and A2B subtypes) on the neural membrane, as well as A3 receptors (A3Rs) [62]. Generally, A1 and A3 adenosine receptors (ARs) lower cAMP levels and inhibit neural activity, while A2A and A2B adenosine receptors promote intracellular cAMP signaling, thus increasing neural activity. An imbalance in the activation of these two receptor groups can result in cognitive impairment and sleep disorders in mice [63,64]. As proposed by Lazarus, these two types of receptors are synchronized during sleep [65]. Pioneering studies have provided compelling evidence of the role of adenosine and its A1 receptor in the regulation of sleep. Multiple lines of evidence suggest that the activation of A1 receptors in mice reduces cholinergic neuronal activity and promotes slow oscillations during NREMS [66,67]. The conditional knockdown of the A1 receptor in CaMKII neurons in mice also alters SWA and interferes with memory formation [68]. Despite this, constitutive A1 receptor knockout mice demonstrate normal SWA and NREMS, suggesting redundant regulation of NREMS [69]. Further, A2ARs govern the transition into the sleep state. A2AR activation in certain brain regions initiates REM sleep and the transition from sleep to wakefulness in mice [70]. However, waking induced by A2AR activation often results in higher-order cognitive deficits akin to those seen after sleep deprivation [71].

Astrocytic cannabinoid type 1 (CB1) receptors have also been reported to modulate neuronal activity through the postsynaptic neurons’ release of endocannabinoid. The latter triggers astrocyte to release glutamate, which then interacts with presynaptic mGluR neuron receptor to regulate the release of presynaptic neurotransmitters [72]. This mechanism, described in various brain regions, is thought to contribute to synaptic plasticity in mice [73]. Astrocytes from the pedunculopontine nucleus (PPN), known to have a role in sleep-wake regulation, exhibit more frequent calcium waves when stimulated by CB1 receptor agonists [74]. Furthermore, Áron Kőszeghy and colleagues demonstrated that astrocyte regulates excitatory and inhibitory postsynaptic currents through endocannabinoid signaling in mice [75]. While a formal link between astrocytic activity and sleep regulation in the PNN is yet to be established, it certainly warrants further investigation.

### 2.3. Astrocytic Ca^2+^

Recent findings demonstrate that astrocyte activity fluctuates in conjunction with sleep and wakefulness, increasing during wakefulness and decreasing during sleep [18,26]. Interestingly, different brain regions may display distinct patterns. For instance, the frontal cortex [26], barrel cortex [18], primary visual cortex [25], hippocampus, and cerebellum have displayed the trend previously mentioned, while the hypothalamus and brainstem have not [27]. With advancements in one/two-photon microscopy and the development of genetically encoded calcium indicators, the astrocyte Ca^2+^ activity could be detected in vivo [76,77]. In vivo studies in mouse models have revealed that sleep-wake cycles are correlated with astrocytic Ca^2+^ activity [18,78]. This implies that astrocyte intracellular calcium concentrations oscillate according to the concentrations of neurotransmitters and neuromodulators. During wakefulness, astrocytes may utilize intracellular calcium oscillations to detect and reinforce nearby neuronal activity [79]. Interestingly, differences in Ca^2+^ levels during NREM/REM sleep have been observed in various brain regions of mice [27]. Attenuation of IP_3_/Ca^2+^ signaling specifically within astrocytes has even been found to cause mice to spend more time in REM sleep [80]. Furthermore, during slow wave sleep (SWS), higher Ca^2+^ signals have been observed in astrocytic processes than in the soma [18]. Astroglial processes enable astrocytes to monitor synaptic activity, and Ca^2+^ activity therein could help maintain CNS homeostasis during sleep, even with their overall reduced activity [81]. Technological improvements in the spatiotemporal detection of Ca^2+^ have uncovered similar dynamic Ca^2+^ patterns within individual astrocytes. Alterations in Ca^2+^ event frequency and amplitude during sleep and wakefulness are localized within different compartments of the cell, with the most significant changes occurring in the processes [26]. Notably, alterations in cerebral cortical astrocytes during sleep-wake transitions are not merely passive responses to peripheral neuronal activity. Cortical neurons exhibited their highest coherence in a mouse model during the slow thalamocortical oscillations characteristic of NREMS, showing a distinct pattern compared to astrocytic Ca^2+^ oscillations [82,83].

Given the oscillation of astrocyte Ca^2+^ activity along the sleep-wake cycle, a model has been proposed wherein astrocytes detect and integrate neuron signals into intracellular Ca^2+^ activity and modulate sleep through negative feedback [12]. Two-photon microscopy has shown that astrocyte Ca^2+^ concentrations align with sleep need, becoming further elevated after sleep deprivation and declining to their lowest levels post recovery sleep [26]. Furthermore, sleep deprivation induces modifications in cortical coherence between astrocytes, as well as in the area and duration of individual Ca^2+^ events [26]. Supporting this, similar research has demonstrated that astrocyte Ca^2+^ levels can change according to sleep need in *Drosophila*. In vivo two-photon imaging also confirmed an increase in astrocytic Ca^2+^ concentrations in the superior medial protocerebrum and antennal lobe in *Drosophila* following sleep deprivation [51].

In light of the associated connection between astrocyte Ca^2+^ concentrations and sleep need, manipulating astroglia Ca^2+^ levels would serve as a method to verify its role in sleep. IP_3_R signaling in astrocytes is crucial for Ca^2+^ signaling. A study found that knockout of IP_3_R2 fragmented NREMS and had more frequent microarousals [18]. However, genetic reduction of IP_3_ in astrocytes in mice did not affect NREMS architecture or NREM SWA, but caused an increase in REM sleep bouts’ frequency [80]. These discrepancies in these results could be due to several factors. Although IP_3_R2 was identified as the dominant subtype, it is worth noting that IP_3_R1 and IP_3_R3 also play a role in facilitating rapid Ca^2+^ events in the processes [84]. The distribution of IP_3_Rs is complex, with IP_3_R2 primarily distributed in the soma and major branches and IP_3_R1 more enriched in peri-synaptic processes [85,86]. Therefore, constitutive knockout of IP_3_R2 may not affect the most dynamic Ca^2+^ signaling in distal processes during sleep-wake cycles. Additionally, the intricate tissue distribution patterns of IP_3_Rs could cause IP_3_R2 knockout to affect sleep-wake behavior beyond its role in CNS astrocytes.

### 2.4. Glymphatic System

Li and coauthors demonstrated that astrocytes play a key role in the clearance of solutes and chemicals from the brain that accumulate during the wake phase. This is accomplished through the formation of glymphatics during sleep [87]. Aquaporin 4 (AQP4) water channels, situated on the astrocytic end-feet in contact with the vasculature, are instrumental in facilitating the flow of cerebrospinal fluid (CSF) from perivascular spaces to the brain parenchyma. Moreover, it has been noted that the extracellular space of the brain expands by approximately 60% during sleep compared to wakefulness [88]. However, recent studies suggest that the regulation of the glymphatic system is predominantly dictated by circadian rhythms, not simply by sleep/wake cycles [89,90]. For instance, when light-dark cycles were reversed in rats, the redistribution pattern of the glymphatic system was found to be similar to that of rats under normal light-dark cycles. This indicates that the function of the glymphatic system is not solely tied to sleep/wake states, but is also affected by endogenous hormones [90]. Further evidence supporting the significant role of astrocytes in sleep regulation is the finding that the distribution of astrocyte end-foot AQP4 polarization correlates with different times of the day. Notably, ablation of the AQP4 gene effectively disrupts the circadian regulation of CSF distribution [91]. Taken together, these findings highlight the pivotal role astrocytes play in regulating sleep and underscore the intricate relationship between sleep, the glymphatic system, and circadian rhythms.

## 3. Potential Role of Astrocytes in Sleep Disorders Comorbidities

The intricate relationship between sleep disorders and other medical conditions, known as comorbidities, presents a complex landscape in medical research. Sleep impairment increases the risk of developing neurodegenerative diseases and psychiatric disorders, and may exacerbate disease pathology and progression. Conversely, patients suffering from various neurodegenerative disorders and other pathological conditions often experience sleep disturbances. Furthermore, many neurodegenerative disorders and pathological conditions, such as pain and epilepsy, frequently coincide with concurrent sleep disturbances. This section provides an overview of these conditions, setting the stage for a deeper exploration into the cellular mechanisms, particularly the role of astrocytes, in their manifestation.

### 3.1. Alzheimer’s Disease

Sleep changes often appear years before the cognitive impairment and clinical manifestations of Alzheimer’s disease (AD), making them potential biomarkers for early detection and intervention [92,93]. Emerging data showed that astrocytes not only modulate sleep but also maintain intracerebral ion and fluid homeostasis, and provide energy to neurons [94]. A cohort study found that sleep fragmentation increased astrocytic activation markers, which was associated with a lower level and faster decline of cognitive function [95]. This finding provided the initial clinical evidence that astrocytes were involved in sleep fragmentation-induced cognitive decline. In fact, a few preclinical studies support this notion. A recent study found that specific knockdown of astrocytic Grin2a reduced autophagy flux, elevated Aβ, and resulted in the deterioration of SD-induced cognitive decline [96]. Interestingly, deletion of astrocytic *Bmal1,* the core circadian clock gene, protected against tau and α-synuclein neuropathologies by induction of Bag3 [97]. Collectively, these data suggest that astrocytes are pivotal in the nexus between sleep disturbances, cognitive decline, and AD pathology, underscoring the importance of their therapeutic exploration.

### 3.2. Parkinson’s Disease

Circadian rhythm and sleep disturbances are notable nonmotor symptoms in Parkinson’s disease (PD) and often manifest before the appearance of typical motor deficits. Emerging research suggests that sleep dysfunction might be a risk factor for PD [98]. The aggregation of α-synuclein is a key neuropathological feature of PD. Notably, during wakefulness, this protein is detected at higher concentrations in the brain extracellular fluid and CSF compared to during sleep, which means astrocyte play a role in the cleaning of α-synuclein [99]. GPR109A, an anti-inflammatory receptor with heightened expression in PD, is present in microglia, suggesting a potential link to neuroinflammation and astrocytes [100]. This interplay between sleep quality, astrocyte and PD underscores the intricate relationship between neuroinflammation, sleep dysfunction, and neurodegeneration in PD.

### 3.3. Multiple Sclerosis

Astrocytes are fundamental to cognitive processes such as memory and learning [101], playing a crucial role in synaptic plasticity and memory consolidation. However, anomalies in astrocyte signaling can result in cognitive impairments, as evident in conditions such as multiple sclerosis (MS) [102]. Additionally, these cells facilitate the glymphatic system, which is responsible for cerebrospinal fluid circulation and waste elimination. It is worth noting that dysfunctions in this system, particularly during sleep disruptions, have been associated with potential neurodegenerative diseases, including MS [103]. Factors such as circadian rhythm disturbances and the release of inflammatory mediators can elevate the MS risk, especially in shift workers [104,105]. To fully grasp the connection between sleep disorders, astrocytes, and MS, more research is imperative, aiming to develop effective interventions for sleep-related challenges in affected individuals.

### 3.4. Huntington’s Disease

Huntington’s disease (HD) patients have been reported to experience a disturbance in their sleep/wake cycle, which manifests early in the disease progression [106]. Evidence suggests that sleep disorders can adversely affect the cerebellar and basal ganglia loops, systems that require appropriate REM sleep and wakefulness to control movement [107]. Although the precise interplay between sleep disorders, shifts in circadian rhythm, and HD is yet to be fully elucidated, recent studies emphasize the significance of astrocytes in deciphering these preliminary nonmotor symptoms. An in-depth analysis of a comprehensive human transcriptomic dataset related to HD pinpointed an astrocyte-specific network in the HD caudate. Intriguingly, this network is intimately associated with sleep and stress characteristics frequently altered in HD patients [108]. This finding underscores the pivotal role of astrocytes in HD, potentially serving as a link between sleep, stress, and the progression of the disease.

### 3.5. Amyotrophic Lateral Sclerosis

Distinct sleep alterations are evident in ALS patients, with motor symptoms such as muscle fasciculations and recurrent cramps intensifying sleep disruptions, particularly at night [109]. Astrocytes, which are integral to the brain’s glymphatic system and are associated with several neurodegenerative diseases, demonstrate diminished functionality in early-stage ALS patients compared to their healthy counterparts [110]. In addition, astrocytes have been reported to show boosted levels of the autophagy signaling protein p62 in ALS patients [111]. Moreover, knocking out reactive astrocyte activating factors markedly extends survival in an ALS mouse model [112]. The effects of sleep dysfunction on astrocytes and their potential involvement in neurodegenerative processes, as well as their role in regulating the wake-sleep cycle in ALS, require further investigation and clarification.

### 3.6. Pain

Sleep deprivation heightens pain sensitivity among healthy individuals. Moreover, chronic pain is associated with the activation of glial cells [113]. Disruptions in the astrocytic glycogen processes, potentially stemming from sleep deprivation, may underlie conditions such as migraines and depression [114]. These disturbances can elevate the brain’s vulnerability to cortical spreading depression due to hindered potassium and glutamate removal [37]. Notably, the glymphatic system, responsible for clearing protein-rich waste from the brain, is influenced by sleep. Malfunctions in this system, potentially arising from astrogliopathy, might compromise the brain’s waste clearance capabilities, resulting in neuroinflammation and sustained pain. These findings highlight the importance of promoting healthy sleep habits and addressing sleep disturbances as part of a comprehensive pain management strategy.

### 3.7. Stroke

Sleep disordered breathing (SDB) stands as an independent risk factor for stroke [115]. Astrocytes, enriched with AQP4, play a pivotal role in preserving brain water equilibrium and are significantly involved in ischemia-reperfusion injuries [116,117]. An imbalance in AQP4 regulation can result in edema, a hallmark symptom of stroke [116]. AQP4 deletion not only amplifies neurological deficits and astrocytic dysfunction but also heightens postischemic inflammation [117,118]. Sleep deprivation boosts the expression of *Neurocan*, an astrocyte-derived gene that impedes growth, predominantly in the ischemia-affected hemisphere [119]. The lncRNA MALAT1 regulates AQP4 expression through miR-145, impacting cerebral injuries [120]. Yet, the precise role of AQP4 in edema is still not well defined, emphasizing the diverse functions of astrocytes in maintaining brain health [116].

### 3.8. Epilepsy

Astrocytes are instrumental in regulating sleep. Notably, during REM sleep in the lateral hypothalamus, they exhibit significant changes, such as a decrease in astrocytic Ca^2+^ and pH, resulting in acidification even with an increased brain-blood volume (BBV) [121]. This acidification, possibly stemming from heightened glutamate transporter activity within astrocytes, underscores their impact on neuronal activity and hints at a potential link between sleep and seizures. The relationship between sleep disturbances and seizures is multifaceted. While sleep deprivation makes certain individuals more susceptible to epileptic seizures [122], research outcomes are inconsistent. Some investigations report no marked rise in epileptic activity due to sleep deprivation [123], whereas others note that sleep deficits can exacerbate seizure episodes [124]. Specific epilepsy syndromes, such as nocturnal frontal lobe epilepsy and juvenile myoclonic epilepsy, have a pronounced association with sleep [125]. Considering the evident reactive astrocytosis in epileptic tissues, which leads to elevated intracellular glutamate concentrations in astrocytes [126], it is conceivable that these cells play a role in the intricate interplay between sleep disruptions and epilepsy.

### 3.9. Traumatic Brain Injury

Traumatic brain injury (TBI) and its milder variant, mild-TBI, profoundly affect sleep-wake patterns, leading to disorders such as insomnia and sleep apnea [127]. Sleep deprivation postmild TBI modifies gene expression in regions such as the cerebral cortex and hippocampus, influencing immunity, inflammation, and glial markers, notably the astrocytic marker GFAP [127]. Elevated serum concentrations of proteins such as GFAP serve as indicators of TBI severity and prognostic markers [128]. Astrocytes, pivotal in the TBI response, modulate processes such as neurogenesis, synaptogenesis, neuroinflammation, and the integrity of the blood-brain barrier. AQP4 in astrocytes is implicated in post-TBI edema, positioning astrocytes as potential therapeutic focal points [129]. Additionally, the glymphatic system is compromised post-TBI. This disruption might facilitate protein buildup, paving the way for neurodegeneration [130].

### 3.10. Postoperative Cognitive Dysfunction

Sleep disturbances, which affect memory consolidation and cognitive abilities, are recognized as an independent risk factor for postoperative cognitive dysfunction (POCD) [131]. Notably, sleep disruption prior to surgery amplifies the risk of postoperative delirium. A study highlighted that patients experiencing sleep disturbances were approximately five times more susceptible to POCD [132]. Furthermore, preoperative deprivation of rapid eye movement sleep exacerbates POCD symptoms following sevoflurane inhalation and triggers astrocyte activation in the CA1 region, underscoring the pivotal role of astrocytes [133]. Astrocytes, interconnected via connexin 43 gap junctions, play a vital role in maintaining cerebral equilibrium. Their functionality, particularly when perturbed by sleep disturbances, can impact various brain areas associated with POCD and perioperative neurocognitive disorders [134]. Given the complex interplay between sleep disturbances, astrocytic functions, and POCD, systematic studies assessing sleep disorder treatments, and their influence on astrocyte activity could provide insights into the direct or indirect contributions of sleep disturbances to POCD.

### 3.11. Anxiety and Depression

Insomnia, being a major predictor of the onset of anxiety [135], has notable connections with anxiety disorders. For instance, insomnia combined with anxiety signals increased morbidity and reduced responsiveness to treatment [136]. Studies have revealed that astrocytes play a pivotal role in instigating neuroinflammation, which has been linked to the onset of mood disorders due to disruptions in the hypothalamic-pituitary-adrenal axis feedback loop [137]. This neuroinflammation, exacerbated by sleep disorders, can lead to atypical synaptic pruning, premature synaptic loss, and neuronal degeneration [138]. Finally, setting the stage for cognitive impairments, memory lapses, and mood disorders, including anxiety.

People suffering from sleep disorders are more likely to develop mental health issues, especially depression. Persistent insomnia acts as an independent risk factor for depression and can lead to unfavorable clinical outcomes [139]. The potential mechanisms between sleep disorders and depression may involve inflammation, disruption of neurotransmitters, genetic correlations, and compromised circadian rhythm [140]. It has been discovered that astrocytes operate by stimulating purine receptors in the medial prefrontal cortex and synaptic ARs [141], leading to antidepressant-like outcomes driven by adenosine.

With a clearer understanding of the comorbidities linked to sleep disorders, it is crucial to identify the cellular players behind these associations. Astrocytes, with their diverse functions, emerge as key figures in this narrative, prompting a deeper exploration in the subsequent section.

## 4. Astrocytes-Mediated Mechanism Underlying Sleep Disorder Comorbidities

**Figure 2:**
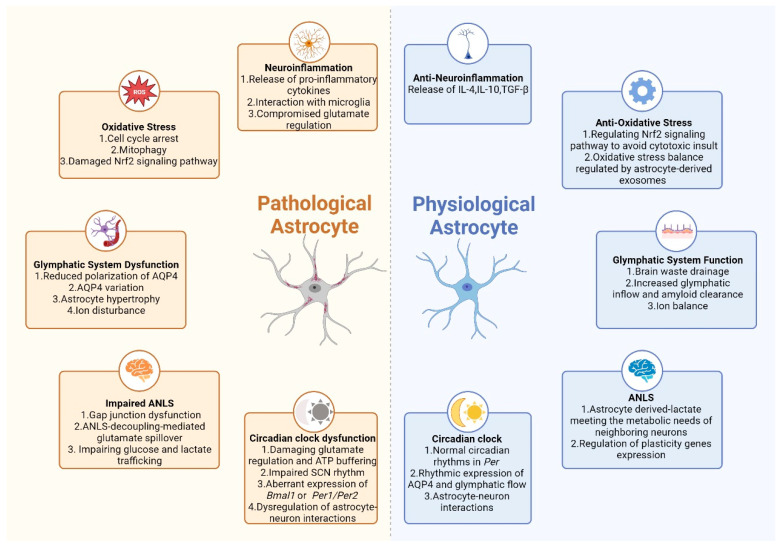
Potential mechanisms of astrocyte in sleep disorders comorbidity. Pathological astrocyte participates in sleep disorders comorbidity via five main mechanisms. (1) Neuroinflammatory signaling activation and compromised glutamate regulation: pathological astrocytes may release pro-inflammatory cytokines or interacting with microglia, triggering an inflammatory response in the brain. Additionally, they may fail to properly uptake and recycle glutamate, leading to an imbalance that affects neural excitability; (2) Aggravated oxidative stress: astrocyte play a part in increased oxidative stress, cell cycle arrest, mitophagy, and Nrf2 signaling pathways; (3) dysfunction of glymphatic system and brain waste drainage: astrocytes, responsible for clearing waste products from the brain, may express AQP4 aberrantly and disrupt ion homeostasis; (4) impaired ANLS resulting in glutamate spillover and unfulfilled metabolic needs for neuron; (5) damaged astrocyte circadian clock function: intrinsic circadian clocks such as *Per1/2* that synchronize with the body’s overall circadian rhythm get impaired in astrocyte, leading to disturbance in astrocyte-neuron interaction.

Astrocytes have emerged as crucial players in the landscape of sleep disorder comorbidity. These cells exert crucial regulatory functions in synaptic plasticity, neuroinflammation, and the maintenance of neurotransmitter homeostasis, all of which are directly implicated in the modulation of sleep quality. Dysregulation in astrocytic signaling has been associated with various sleep disorders as well as their comorbidities such as anxiety, depression, and neurodegenerative diseases (Figure 2). Elucidating the intricate interplay between astrocytes and sleep disorder comorbidity holds substantial promise for advancing the understanding of these multifaceted conditions.

### 4.1. Neuroinflammation

Astrocytes, known for their vital role in the innate immune response, contribute diversely to the pathology of sleep disorder comorbidity. In particular, astrocytes react to inflammatory signals and may contribute to the development of comorbid conditions associated with chronic inflammation. Sleep homeostasis disruptions can lead to peripheral inflammation, which can impact the brain in different ways. Subsequently, such stimuli could activate astrocyte, and in turn, activated astrocytes released proinflammatory cytokines and interacted with microglia, which probably amplified neuroinflammatory response in the brain [142]. Astrocytes, similar to microglia, demonstrate heterogeneity dependent on the characteristics of the stimuli [143]. An increase in proinflammatory factors such as IL-1β, TNF-α, and NO, signifies proinflammatory phenotype in astrocytes [144]. In sleep-deprived mice, astrocytes have been found to produce NLRP3, facilitating IL-1β maturation. Moreover, chronic sleep deprivation was found to increase the cortical expression of phagocytic markers, such as Mertk, which localized on astrocytes [145]. Contrastingly, neuroprotective astrocytes release anti-inflammatory cytokines, such as IL-4, IL-10, and transforming growth factor-β (TGF-β) [146]. It is notable that sleep deprivation is linked to the activation of nuclear factor kappa-light-chain enhancer of activated B cells (NF-κB) in the cerebral cortex of mice [147]. Importantly, NF-κB activation promotes the expression of IL-1 and TNF [148], which can further stimulate astrocytic NF-κB, ultimately triggering neuronal death [149].

Inflammation is considered as a fundamental and common pathogenic factor in sleep disorder comorbidity due to the universal observation of inflammatory activation under these conditions. In the context of sleep disorders, growing evidence suggests a reciprocal relationship between astrocyte-mediated neuroinflammation and sleep disturbances. For instance, enhanced astrocyte activation and elevated proinflammatory cytokine levels have been documented in patients with OSAS [150]. Likewise, neurodegenerative disorders, including AD and PD, frequently present sleep disturbances. In these conditions, astrocyte activation and the ensuing release of inflammatory mediators can exacerbate neuronal damage, synaptic dysfunction, and cognitive decline [151]. The complex interplay between astrocyte-mediated neuroinflammation and sleep disorders warrants a deeper understanding due to its significant clinical implications.

### 4.2. Oxidative Stress

Disturbances in sleep can elevate levels of oxidative stress, given the crucial role of sleep in redox homeostasis and repairing oxidative damage to tissues. A study found that when faced with increasing oxidative stress, reactive human astrocytes exhibit a senescence phenotype marked by rising levels of p16, p21, p53, and G1 cell cycle arrest [152]. Furthermore, oxidative stress disfunction in aging-related CNS disorders such as AD and PD may comprise a common neurophysiologic foundation for lesion and promote the aging-degenerative process. Importantly, astrocytes play a neuroprotective role by upregulating the nuclear factor erythroid 2-related factor 2 (Nrf2) signaling pathway, which shields them from cytotoxic insults [153]. Additionally, the upregulation of Nrf2 signaling and increased levels of HO-1 have been observed to counterbalance elevated levels of oxidative stress in PD and AD [154]. Notably, astrocyte activation has also been noted in ALS patients, where it may significantly impact the progression of ALS by mediating the SQSTM1/p62-Nrf2 antioxidative stress pathway [155]. Dysfunctional astrocytes leading to oxidative stress can disrupt normal sleep-wake regulation, thereby perpetuating sleep disturbances and potentially exacerbating the severity and progression of associated comorbid conditions.

In a study utilizing a spinal cord injury mouse model to investigate chronic neuropathic pain syndrome, researchers found that knocking down the receptor expressed on myeloid cells 1 (TREM1) resulted in reduced astrocyte activation and oxidative stress levels [156]. Additionally, other studies have revealed that inhibiting astrocytic GFAP expression after nerve injury could relieve neuropathic pain behaviors [157]. Astrocytes have also been found to express transient receptor potential ankyrin 1 (TRPA1), a potential player in pain progression. Moreover, astrocyte-derived exosomes are reported to protect against TBI-induced oxidative stress by activating Nrf2 signaling in rodents [158]. Notably, mice with brain hemorrhage exhibited increased peroxynitrite production, leading to oxidative damage and depleted GSH levels in astrocytes [159]. Focusing on the mechanisms involved in astrocytic oxidative stress may lessen its detrimental effects on sleep regulation and lighten the burden of associated comorbidities.

### 4.3. Impaired Glymphatic System

Sleep is known to remove harmful molecules from the brain by regulating the glymphatic system. This system, formed by astrocytes, is believed to enhance brain waste drainage. Notably, some AQP4 single nucleotide polymorphisms (SNPs) have been associated with reduced self-reported sleep quality and an elevated amyloid load in individuals. This implies that AQP4 variation may influence both amyloid accumulation and sleep quality [160]. Further, both animal and clinical studies have confirmed that slow waves, characteristic of NREM sleep, correspond with increased glymphatic inflow and amyloid clearance. This reinforces the idea that deep sleep is especially essential for brain clearance [161,162]. Furthermore, TAR-DNA-binding protein 43 (TDP-43), the protein most significantly associated with ALS, has been observed to accumulate in instances of compromised glymphatic flow and AQP4 depolarization [163]. The pathological presence of gliosis in ALS, marked by astrocyte hypertrophy and microglial proliferation, further suggests potential glymphatic system damage [164].

TBI in individuals with comorbid sleep disorders can compromise the glymphatic system, leading to significant biomarker accumulation in the brain [165]. Furthermore, the recovery duration of AQP4 polarization distribution correlates positively with TBI severity [166]. Additionally, acute hypoxia has been found to induce AQP4 localization in cortical astrocytes, subsequently increasing water permeability [167]. Consistently, studies have shown that CSF enters the brain through the glymphatic pathway, leading to early-stage ischemic stroke edema formation and ion disturbances [168]. Moreover, chronic unpredictable mild stress exposure in a mouse model has shown a decrease in AQP4 expression in the cortex and hippocampus. This has been accompanied by widespread loss and distribution of AQP4 in astrocytes, suggesting glymphatic system disruptions in animal models of depression [169,170]. Similar trends were observed in the brains of patients with major depressive disorder (MDD), with AQP4 expression found to be downregulated in the locus coeruleus and hippocampus [171,172].

### 4.4. Astrocyte-Neuron Lactate Shuttle

One crucial function of astrocytes is to regulate the metabolic demands of neurons through a mechanism called the ANLS, which is closely tied to the sleep/wake cycle [173]. Lactate derived from glycolysis in astrocytes works as an important energy substrate to meet the metabolic needs of neighboring neurons. It modulates mechanisms governing synaptic plasticity and memory consolidation by regulating plasticity gene expression [174,175]. Studies have demonstrated that sleep deprivation can upregulate cortical astrocyte genes associated with the ANLS process, suggesting that astrocytes play a critical role in linking neurometabolic coupling to the sleep/wake cycle [176]. A recent study reported a correlation between brain lactate levels and glymphatic clearance in mice. When the gap junction subunit, connexin 43, is knocked down in astrocytes, it disrupts glucose and lactate trafficking, which in turn silences wake-promoting neurons. This chain of events leads to excessive sleepiness and fragmented wakefulness in mice [177]. Astrocyte retraction from synapses might result in neuronal damage due to glutamate toxicity. Concurrently, ANLS decoupling could potentially lead to glutamate spillover, promoting increased excitability, excitotoxicity, and neurodegeneration in the surrounding synapses. Reports indicate that Aβ can trigger glutamate release from astrocytes, leading to synapse loss and altering the metabolic functions of astrocytes [178]. These findings prove that a vicious cycle of wakefulness-induced Aβ release, aggregation, and ANLS uncoupling may exacerbate AD progression.

Interestingly, the glial pathology of mood disorders diverged from that observed in neurodegenerative diseases, with the absence of astrogliosis suggesting that dysfunctional astroglia may contribute to the underlying pathophysiology of mood disorders [179]. Sleep disturbances may contribute to the pathophysiology of mood disorders by impeding glycogen turnover, thereby reducing the energy supply to glutamatergic synapses through ANLS. Studies have demonstrated that inhibition of connexin 43 exacerbates depression-like behavior [180]. Supporting these findings, further research has indicated that the gap junction channels of astrocytes could potentially serve as targets for new antidepressant drug development [181,182]. Approaches such as enhancing astrocytic lactate production or promoting lactate uptake by neurons could potentially restore energy balance, improve sleep quality, and ameliorate comorbid conditions.

### 4.5. Dysregulated Astrocytic Circadian Clock

Circadian rhythms play a crucial role in regulating molecular mechanisms that respond to injury and illness. Disruptions in circadian rhythms can negatively impact cognition, immunity, and the overall patient’s recovery [183]. Sleep disorders often include various degrees of circadian disturbances, and the dysfunction of the astrocyte circadian clock may contribute to several pathophysiological processes. In the context of AD, disturbances in the astrocyte circadian clock lead to disruptions in the rhythmic expression of AQP4, resulting in the impairment of glymphatic flow [184]. This dysfunction may also impair glutamate regulation and ATP buffering, making neurons more susceptible to additional insults. Specific genetic changes, such as astrocyte-specific *Bmal1* knockout, have been found to disturb GABA signaling, leading to impaired behavioral arrhythmia [185]. A recent study has also highlighted the variable gene expression of the primarily astrocytic chitinase-3-like protein 1 (*Chi3l1* or YKL-40) in AD patients. This variation can result in decreased CSF YKL-40 expression, potentially leading to better cognitive performance over extended periods. In a mouse APP/PS1 model of AD, the deletion of *Chi3l1* was found to enhance the periplaque microglia activation and reduce amyloid plaque accumulation. Interestingly, the expression level of astrocytic core clock proteins like *Bmal1* or negative clock regulators *Per1/Per2* may regulate *Chi3l1* expression. This connection implicates the astrocyte clock in inflammation, phagocytic activation, and AD pathogenesis [186]. Disruption of astrocyte circadian rhythm may also be linked to the inadequate activation of Nrf2 and the deregulation of astrocyte-neuron interactions within and outside the suprachiasmatic nucleus (SCN). Interactions between astrocytes and neurons can shield neurons from iron-mediated toxicity. However, once compromised, dopaminergic neurons become more susceptible to oxidative damage and ferroptosis, leading to cell death [187]. Epidemiological evidence has demonstrated a link between circadian misalignment/shift work and neurodegeneration [188,189]. Thus, it is plausible to hypothesize that impairment of astrocytes and their clocks may also contribute to this phenomenon.

## 5. Limitations

While this study provides promising insights into the role of astrocytes in regulating sleep and its associated comorbidities, several limitations should be clarified. First, the complexity of astrocyte-mediated mechanisms in sleep regulation is vast, and our understanding is still in its infancy, with the specificity of some somnogenic molecules and astrocyte-associated receptors varying across different species and physiological conditions. Second, the majority of the evidence presented relies on in vivo studies, which may not fully represent the in vitro environment, limiting the generalizability of our findings. Third, the intricate relationship between sleep disorders and comorbidities, such as neuroinflammation, oxidative stress, and impaired glymphatic system, is multifaceted, and our study may not encompass all potential interactions and underlying mechanisms. Last, but not the least, some of the methodologies employed in assessing astrocytic Ca^2+^, ANLS, and dysregulated astrocytic circadian clocks may have inherent biases or inaccuracies. The development of more refined and standardized techniques would enhance the reliability of future studies.

## 6. Conclusions

Understanding the role of astrocytes in sleep regulation is essential for advancing our knowledge of both normal sleep patterns and sleep disorders. In a healthy brain, astrocytes play a vital role in regulating the sleep-wake cycle through specific processes at the molecular level. They control the release and uptake of somnogenic molecules such as adenosine, lactate, and serotonin, which are key to maintaining sleep homeostasis. The distinction between functional and dysfunctional astrocytes becomes evident in pathological conditions. Dysfunctional astrocytes may contribute to sleep disturbances, acting as both a causal and worsening factor in neurodegenerative diseases and other health issues. This dysfunction can manifest in various ways, including neuroinflammation, oxidative stress, glymphatic system dysfunction, disrupted ANLS, and a dysregulated astrocytic circadian clock. The goal of our research is to thoroughly characterize these specific pathological changes to facilitate more effective therapeutic interventions. We aim to answer critical questions such as how astrocytes regulate non-REM sleep and REM sleep, and what are the underlying molecular mechanisms in both normal and pathophysiological conditions. Addressing these questions will pave the way towards a better understanding of the increasingly complex role of astrocytes in the brain. This understanding underscores the need for further studies and will guide the development of targeted therapies for comorbidities related to sleep disorders.

## Abbreviations

AD	Alzheimer’s disease
ALS	Amyotrophic lateral sclerosis
ANLS	astrocyte-neuron lactate shuttle
AQP4	Aquaporin 4
ARs	adenosine receptors
ATP	adenosine triphosphate
BBB	blood-brain barrier
BBV	brain-blood volume
CB1	cannabinoid type 1
cAMP	cyclic adenosine monophosphate
CNS	central nervous system
CSF	cerebrospinal fluid
DREADDs	designer receptors exclusively activated by designer drugs
dnSNARE	dominant-negative SNARE
EEG	electroencephalogram
ER	endoplasmic reticulum
GHRH	hormone-releasing hormone
GPCRs	G-protein-coupled receptors
HD	Huntington’s disease
IL-1	Interleukin-1
IL1R1	Interleukin-1 receptor 1
IP_3_R2	IP_3_-activated type 2 receptors
MDD	major depressive disorder
MS	multiple sclerosis
mPFC	medial prefrontal cortex
NF-κB	nuclear factor kappa-light-chain-enhancer of activated B cells
NREM	nonrapid eye movement
NREMS	nonrapid eye movement sleep
Nrf2	nuclear factor erythroid 2-related factor 2
OPCs	Oligodendrocyte precursor cells
OSAS	obstructive sleep apnea syndrome
PD	Parkinson’s disease
PPN	pedunculopontine nucleus
REM	rapid eye movement
RBD	REM behavioral disorder
RLS	restless legs syndrome
SCN	suprachiasmatic nucleus
SDB	sleep disordered breathing
SNPs	single nucleotide polymorphisms
SWA	Slow-wave activity
SWS	slow-wave sleep
TBI	Traumatic brain injury
TDP-43	TAR-DNA-binding protein 43
TGF-β	transforming growth factor-β
TNFα	tumor necrosis factor α
TRPA1	transient receptor potential ankyrin 1
TREM1	triggering receptor expressed on myeloid cells 1
